# Radiomics-Assisted Computed Tomography-Based Analysis to Evaluate Lung Morphology Characteristics after Congenital Diaphragmatic Hernia

**DOI:** 10.3390/jcm12247700

**Published:** 2023-12-15

**Authors:** Silviu-Viorel Virlan, Matthias F. Froelich, Greta Thater, Neysan Rafat, Julia Elrod, Michael Boettcher, Stefan O. Schoenberg, Meike Weis

**Affiliations:** 1 Department of Clinical Radiology and Nuclear Medicine, University Medical Center Mannheim, Theodor-Kutzer-Ufer 1–3, 68167 Mannheim, Germany; matthias.froelich@umm.de (M.F.F.); greta.thater@umm.de (G.T.); stefan.schoenberg@umm.de (S.O.S.); 2 Department of Neonatology, Center for Children, Adolescent and Women’s Medicine, Olgahospital, Clinic of Stuttgart, 70174 Stuttgart, Germany; n.rafat@klinikum-stuttgart.de; 3 Department of Pediatric Surgery, University Medical Center Mannheim, Theodor-Kutzer-Ufer 1–3, 68167 Mannheim, Germany; julia.elrod@umm.de (J.E.); michael.boettcher@medma.uni-heidelberg.de (M.B.)

**Keywords:** congenital diaphragmatic hernia, radiomics, ECMO, pulmonary hypoplasia

## Abstract

**Purpose:** Children with congenital diaphragmatic hernia suffer from long-term morbidity, including lung function impairment. Our study aims to analyze lung morphology characteristics via radiomic-assisted extraction of lung features in patients after congenital diaphragmatic hernia repair. **Materials and Methods:** 72 patients were retrospectively analyzed after approval by the local research ethics committee. All the image data were acquired using a third-generation dual-source CT (SOMATOM Force, Siemens Healthineers, Erlangen, Germany). Dedicated software was used for image analysis, segmentation, and processing. **Results:** Radiomics analysis of pediatric chest CTs of patients with status after CDH was possible. Between the ipsilateral (side of the defect) and contralateral lung, three shape features and two higher-order texture features were considered statistically significant. Contralateral lungs in patients with and without ECMO treatment showed significant differences in two shape features. Between the ipsilateral lungs in patients with and without the need for ECMO 1, a higher-order texture feature was depicted as statistically significant. **Conclusions:** By adding quantitative information to the visual assessment of the radiologist, radiomics-assisted feature analysis could become an additional tool in the future to assess the degree of lung hypoplasia in order to further improve the therapy and outcome of CDH patients.

## 1. Introduction

Congenital diaphragmatic hernia (CDH) is a disease with an incidence of 1 in 2000 to 5000 births, up to this date without a clear origin of pathogenesis, but with multiple theories including exposure to teratogens such as the herbicide nitrofen [1], genetic factors with a multifactorial inheritance mode based on the phenotypic and genetic heterogeneity of CDH [2], certain pharmacological agents like phenmetrazine, quinine, thalidomide, or even vitamin A deficiency [2,3].

CDH can be classified into several types: Posterolateral hernias (Bochdalek hernias) comprise approximately 80%–90% of all CDH, and non-posterolateral (non-Bochdalek) hernias include Morgagni (ca. 2%), central (ca. 2–5%), other anterior hernias associated with the pentalogy of Cantrell, and bilateral hernias (associated with a poor prognosis). About 85% of Bochdalek hernias occur on the left side, about 10% on the right, and approximately 5% are bilateral [4,5].

CDH is associated with high perinatal mortality and long-term pulmonary morbidity, the main contributing factors being lung hypoplasia and persistent pulmonary hypertension of the newborn (PPHN) due to the abnormal morphology of the pulmonary vasculature. These lead to perinatal severe respiratory insufficiency in over 90% of cases [6].

In a study published in 2010, it was reported that survival rates for individuals with congenital diaphragmatic hernia (CDH) were 64.6% at 1 week, 58.4% at 1 year, and 57.1% at 20 years [7]. Notably, patients requiring extracorporeal membrane oxygenation (ECMO) may experience a decrease in survival rates, potentially falling as low as 50% [8]. However, specialized centers have demonstrated significant enhancements in survival rates for ECMO candidates, achieving levels ranging from 85% to an impressive 96% [8]. These improvements are attributed to the implementation of standardized treatment protocols, emphasizing optimized ECMO criteria and lung-protective ventilation strategies [8]. The heightened focus on enhancing survival rates for CDH patients has subsequently directed attention toward addressing long-term morbidities associated with the condition [8]. These morbidities manifest across multiple systems, necessitating comprehensive and multidisciplinary follow-up approaches [9].

Some of the most commonly encountered forms of morbidity in survivors of congenital diaphragmatic hernia (CDH) encompass bilateral lung hypoplasia, ventilator-induced lung injury and hyperinflation, diaphragmatic dysfunctions, as well as anatomical alterations of the spine and thoracic wall [9]. The persistence of pulmonary hypertension is noted, with an incidence ranging between 3% and 12%, and it has been demonstrated to resolve within weeks up to months following surgery, with some reports suggesting persistence into adolescence [10]. The vast majority of survivors of CDH tend to develop gastroesophageal reflux disease (GERD) due to the intricate interplay of mechanical and developmental factors, such as a short abdominal esophagus, developmental impairment of sphincter function, or an excess of abdominal pressure across the hiatus in the postoperative period [11].

Complications such as pneumonia, asthma, or pneumothorax arise in a substantial percentage of cases, ranging from 10% to 50% [10]. Notably, chronic pneumonia, manifesting recurrently throughout the year, stands out as the most prevalent concern, affecting up to 8.8% of survivors who may necessitate hospitalization by the time they reach 1.5 years [10]. Some patients were also shown to develop pneumothorax [10].

A comprehensive examination of 747 patients conducted by Yamoto et al. in 2022 has yielded insightful findings regarding the recurrence rates of congenital diaphragmatic hernia (CDH). According to the study, recurrence rates were reported as 4.3% by the time of discharge, 7.2% between discharge and 1.5 years old, 2.1% between 1.5 and 3 years old, and 1.9% after reaching 3 years old [10]. Notably, the utilization of prosthetic patches in treating CDH has been identified as a predictor of poor outcomes, as highlighted in various studies [9,12,13]. These patches are typically employed in cases involving a large defect and have been associated with compromised lung mechanics and impaired diaphragmatic function, along with an elevated risk of adhesive intestinal obstruction [9,10]. In contrast, when suturing is employed, the recurrence rate is approximately 4%, whereas patch repair significantly escalates the risk, reaching as high as 46% [10,14].

Pulmonary hypoplasia represents one of the most important factors that influence morbidity and survival rates [15]. The extent to which a correlation exists between pulmonary hypoplasia and diaphragmatic defects remains a topic not entirely elucidated [16]. Older studies performed on fetal rabbits and sheep showed that the lack of fetal breathing movements, induced by spinal transection above the phrenic motoneurons without altering the normal diaphragm, led to lung hypoplasia [17]. In these particular studies, the lungs exhibited not only a reduction in size and volume but also structural immaturity characterized by poorly expanded terminal sacs, thicker walls, and diminished compliance when expanded [17]. The halting of alveolar development at the mid-canalicular stage is a widely observed occurrence in congenital diaphragmatic hernia (CDH) [18].

In a study from 2010, Richard Keijzer et al. postulated a dual-hit hypothesis of the pathogenesis of CDH, which explains the pulmonary hypoplasia as a result of two developmental insults. One is occurring before the closure of the diaphragm in utero, based on genetic and environmental factors. The other affects only the ipsilateral lung as a result of the herniation of the abdominal organs into the thorax [19]. These factors are postulated to be responsible for lung hypoplasia, reduced airway branching, surfactant deficiency, and persistent pulmonary hypertension.

Besides the short-term benefits, the use of Extracorporeal Membrane Oxygenation (ECMO) therapy in CDH patients is associated with relevant complications such as bleeding. ECMO is recommended on clinical grounds as a means to stabilize the newborn in the context of severe but potentially reversible respiratory failure prior to surgical repair [20]. Patients with CDH have the longest extracorporeal life support duration and the lowest survival rates of all neonates requiring ECMO [21]. The 2015 updated CDH EURO Consortium Consensus recommends surgical repair of the defect in the diaphragm be performed after physiological stabilization; repair while the patients are on ECMO is also a viable option [6].

Lung impairment correlates with morbidity, survival, and the need for ECMO therapy [22]. Consequently, status after ECMO therapy can be regarded as a surrogate for higher disease severity, as performed previously [23].

In high-volume centers, follow-up of CDH children has been introduced in order to monitor and evaluate therapeutic options for morbidity, including pulmonary morbidity. It has been demonstrated that CT lung density is significantly reduced on the ipsilateral side [24]. One important contributing factor to the development of the emphysema-like changes is the impairment of alveolar multiplication with consecutive overexpansion of the existing alveoli that has been previously observed at autopsies [25].

Initially, radiomics has been used in oncology studies; however, it has the potential to be applied to all diseases [26]. Radiomics is defined as “the high throughput extraction of quantitative imaging features or texture (radiomics) from imaging to decode tissue pathology and create a high dimensional data set for feature extraction” [27]. The purpose of radiomics is to find hidden information within radiological images and extract it using advanced texture and shape analysis [28]. Radiomics extracts abstract textural parameters, which, in simplified words, are a mathematical representation of adjectives such as rough, course, smoots, etc., and could theoretically be applied to any image that could be described as such [29].

Radiomics has the capacity to analyze a vast amount of structural information regarding the lungs. With our study, we aim to extract and select the relevant radiomic features in order to characterize lung morphology characteristics after CDH repair. In the future, these features may be used for advanced prognosis and treatment.

## 2. Materials and Methods

### 2.1. Patients

For this study, 99 CT scans of patients with CDH were identified. Twenty-seven were excluded because they were scanned by other CT scanners in our institution; the image quality was suboptimal, showing large numbers of artifacts, and one of them had an associated malformation that could interfere with the lung-parenchyma measurements.

Finally, we included a group of 72 patients of ages between 2 months and 18 years (with a standard deviation of 51.9 months and a mean deviation of 36.3 months), the inclusion criteria being CDH-repair receiving follow-up controls via a chest CT examination. The patients were selected between 2015 and 2021 from our institute. The patients were examined based on the standardized follow-up protocols of the university medical center Mannheim, Heidelberg University (Germany), one of the national referral centers for CDH. 22 out of the 72 included patients (30.5%) underwent ECMO therapy during the neonatal period. A total of 49 patients had left-side hernias, 22 had right-side hernias, and 1 had bilateral hernias.

### 2.2. Imaging Protocols

For this study, a third-generation dual-source CT (SOMATOM Force, Siemens Healthineers, Germany) was used. All examinations were made using the same Protocol, in accordance with previous studies and guidelines regarding pediatric thoracic low dose imaging, as follows: a tube voltage of 100 kVp with automated tube current modulation with additional tin filtering (0.6-mm tin filter adjacent to the source), a Gantry rotation time of 0.25 s, a pitch of 3.2, and a detector collimation of 2 × 192 × 0.6 mm. A topogram with 80 kVp/34 mA was executed to plan the scan area.

### 2.3. Image Reconstruction

The images were reconstructed using advanced modeling iterative reconstructions [ADMIRE] (Siemens Healthineers, Germany) and a slice thickness of 1.5 mm. The reconstructions used dedicated lung as well as soft tissue convolution kernels; however, for our measurements in this study, we have selected the lung convolution kernel Bl57.

### 2.4. Segmentation and Analysis Software

Image analysis and segmentation were made using the image analysis software Syngo.via Client version 8.3 (syngo.via, Siemens Healthineers, Germany). The patient’s lungs were segmented and measured automatically, with the results being visually corrected and adjusted where necessary, for example, in situations where the lung contours were not exactly identified by the software algorithm.

### 2.5. Image Analysis and Segmentation

The following steps were taken during the lung segmentation: (1) segmentation and separation of the left and right lung based on Hounsfield Unit value recognition of parenchyma; (2) trachea extraction; and (3) lung region refinement.

### 2.6. Feature Extraction

Features were calculated using feature descriptors to quantify characteristics of the gray levels within each region of interest (ROI) or volume of interest (VOI) [30]. Using dedicated software (pyradiomics, version 3.0.1, an imaging biomarker standardization initiative definition-based Python package), features were extracted and further statistically analyzed. The following original radiomic features were extracted and analyzed: “firstorder,” describing the distribution of Hounsfield intensities without comparing to the spatial reference; “shape,” describing the 3D and 2D shapes of the region of interest (ROI); and higher-order texture features, of which “glrlm” [Gray Level Run Length Matrix] and “gldm” [Gray Level Dependence Matrix] are textural features that describe the distribution of gray tones calculated by statistical comparison with the surrounding voxels [27].

### 2.7. Clustering, Feature Selection, and Statistical Analysis

All analyses were performed in R statistics (version 4.1.0, R: A language and environment for statistical computing R Foundation for Statistical Computing [31]). All demographic and clinical parameters were summarized with median and interquartile range (IQR). For the analysis, the packages “tableone,” “Boruta,” “dplyr,” “ggplot2,” “ggcorrplot,” and “caret” were utilized [31].

The Boruta Package performs a shuffled copy of the predictor values and joins them with the original predictors [32,33]. A random forest is built on the merged dataset. Then a comparison of the original variables with the randomized variables is executed in order to assess their importance [32,33]. The variables that are considered important are only the ones with higher importance than those of the randomized variables [32,33].

For feature selection, we used permutation-based random forest (RF) feature selection with the Boruta package. Non-reproductible features were excluded.

The remaining radiomic features were normalized according to the following formula [31]:

$$z=\frac{\left(X-\mu \right)}{\sigma }$$

According to this formula, each feature value *X* was scaled with the mean (*µ*) and standard deviation (*σ*) of the lesion features [31].

The following step involved a feature-to-feature correlation, for which the Pearson correlation coefficient was used. This unsupervised clustering of all radiomic features was viewed as a heatmap [31]. Only one representative feature per correlation cluster was selected.

A *p*-value lower than 0.05 was regarded as statistically significant. Following the Boruta RF-feature selection, the most important variables were analyzed for significant differences by means of an unpaired two-tailed *t*-test [31].

**Figure 1:**
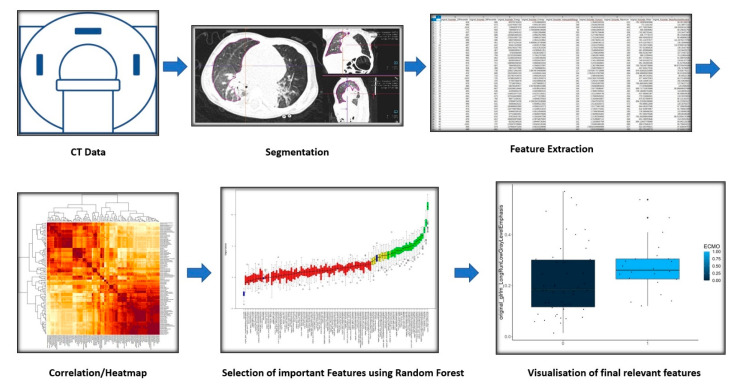
Schematic depiction of the Radiomics process for extracting relevant features.

Using a Pearson correlation threshold in R, we have identified and eliminated a number of redundant features. The selected final features were visualized in boxplot diagrams. Figure 1 visually summarizes the radiomics pipeline.

## 3. Results

### 3.1. Patient Collective and Extracted Features

A total of 72 patients were enrolled in our study. Twenty-two out of the 72 patients (representing 30.5%) underwent ECMO therapy at birth. Forty-nine of the patients had the diaphragmatic hernia on the left side, twenty-two on the right, and one had a hiatus hernia affecting both sides.

In total, our analysis extracted 110 features from each lung that have been further evaluated. The quantitative values for each feature can be found in Supplementary Materials.

### 3.2. Cluster Analysis

**Figure 2:**
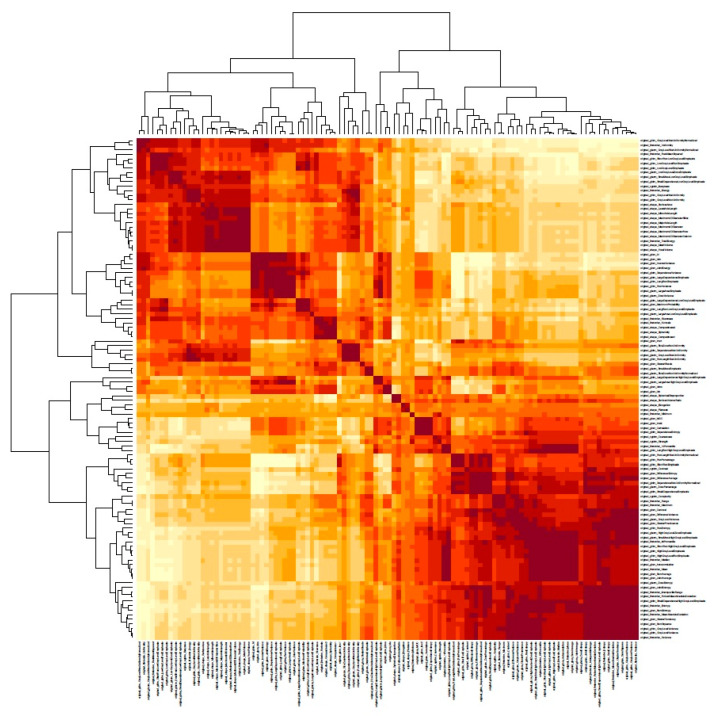
Visual sample of the Pearson correlation coefficient matrix with unsupervised clustering of all radiomic features viewed as a heatmap with feature-to-feature correlation.

After extraction and standardization, an unsupervised hierarchical clustering of features extracted from the lungs was performed, and a heatmap of the features was created, as seen in Figure 2.

### 3.3. Variable Importance Assessment

**Figure 3:**
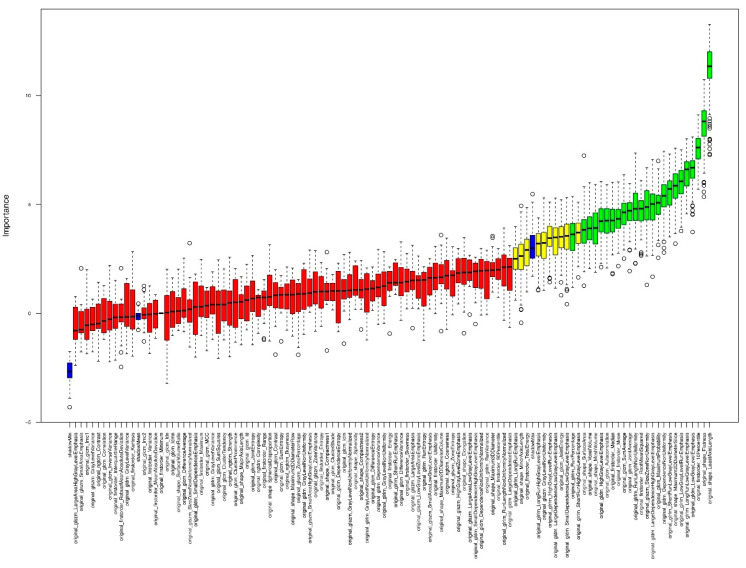
Visual sample of the plot of Boruta feature selection process for the **contralateral lung vs. the ipsilateral lung** outcome. Blue = reference levels during the run of Boruta algorithm; green = significantly different variables between the two lungs (ipsilateral vs. contralateral); yellow = uncertain variables; red = not relevant variables.

**Figure 4:**
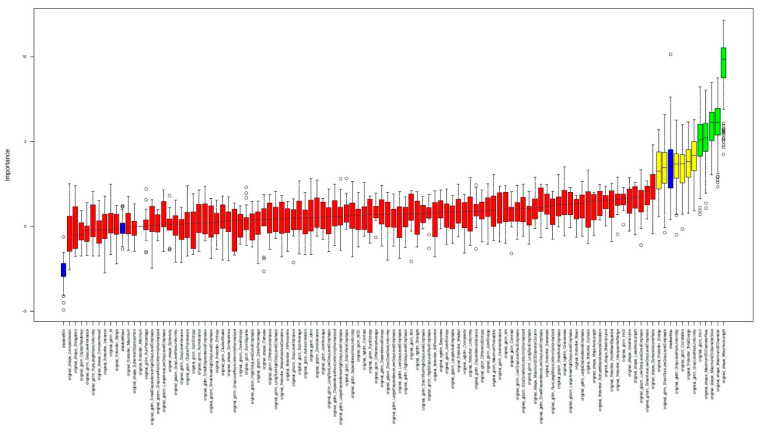
Visual sample of the plot of Boruta feature selection process for significant differences between the **contralateral lungs** in patients with and without ECMO treatment. Blue = reference levels during the run of Boruta algorithm; green = significantly different variables between the contralateral lungs; yellow = uncertain variables; red = not relevant variables.

In order to identify important features concerning our endpoints “ipsilateral vs. contralateral,” “ECMO-contralateral vs. non-ECMO-contralateral,” and “ECMO-ipsilateral vs. non-ECMO-ipsilateral,” we used a random forest (RF) classification algorithm, specifically the Boruta algorithm of the Boruta R Package. Figure 3 and Figure 4 show the important, significantly different features as noted in their descriptions.

Details of all significant features on the obtained sets are included in our Supplementary Materials.

### 3.4. Reduction of Feature Redundancy and Final Feature Selection

**Figure 5:**
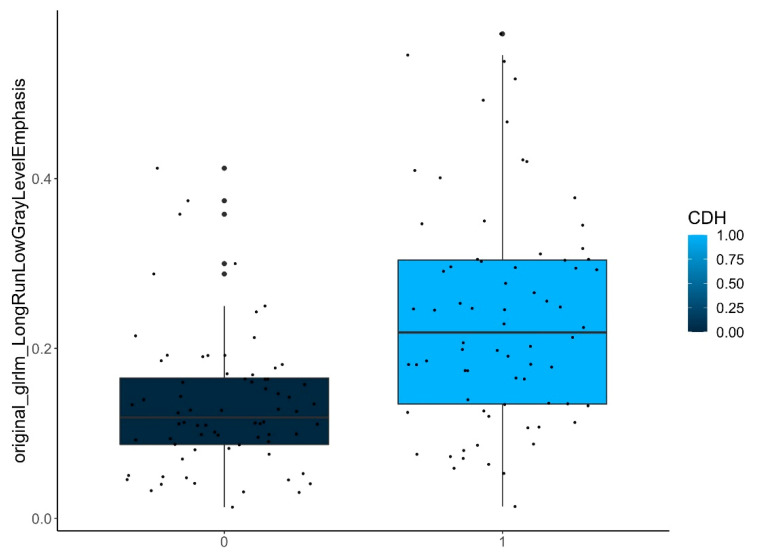
Difference of a selected feature (original_glrlm_LongRunLowGrayLevelEmphasis) between ipsilateral (1) and contralateral (0) lungs.

As previously stated, only one representative feature per correlation cluster was selected. Using a Pearson correlation threshold in R Studio, we have identified and eliminated a number of redundant features. The selected final features were visualized in boxplot diagrams. The following features remained relevant:

A. Differences between the ipsilateral and contralateral lungs:

Here, three shape features, one first-order feature, and two higher-order texture features were considered statistically relevant (Table 1 and Figure 5).

**Figure 6:**
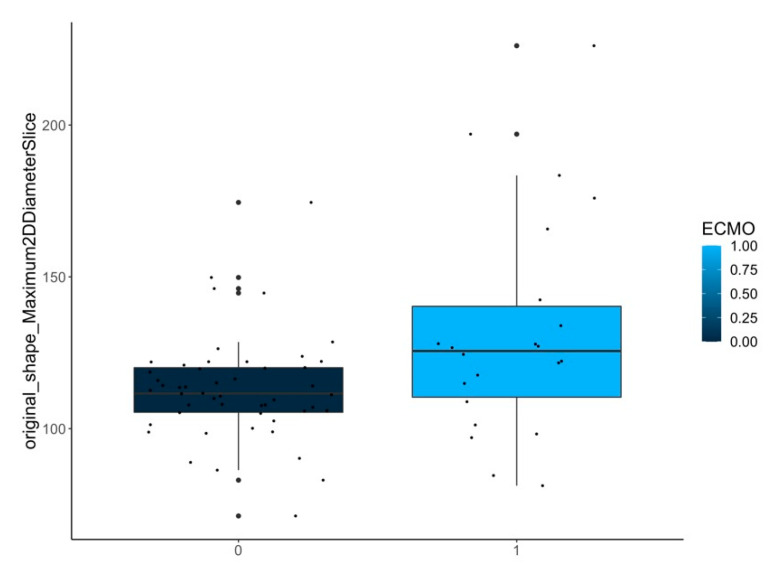
Difference of a selected feature (original_shape_Maximum2DDiameterSlice) between the **contralateral side** in patients with (1) and without (0) need of ECMO treatment.

B. Differences between the contralateral lungs in patients with and without ECMO treatment:

Here, two shape features were considered to be statistically relevant (Table 1 and Figure 6).

**Figure 7:**
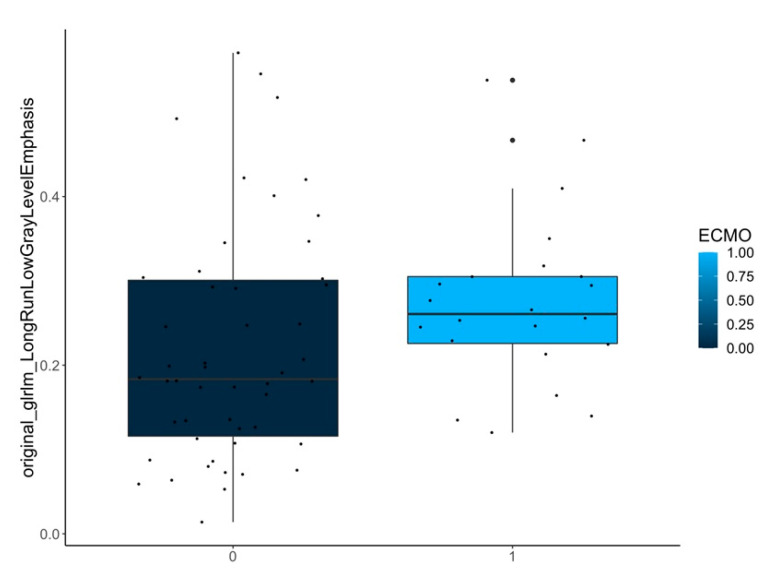
Difference of a selected feature (original_glrlm_LongRunLowGrayLevelEmphasis) between the **ipsilateral lungs** in patients with (1) and without (0) need of ECMO treatment.

C. Differences between the ipsilateral lungs in patients with and without ECMO treatment:

Here, a higher-order texture feature was selected as statistically relevant (Table 1 and Figure 7).

Figure 5, Figure 6 and Figure 7 show samples of boxplots with relevant features.

## 4. Discussion

Our study demonstrates that radiomics analysis of pediatric low-dose chest CT after CDH is possible and relevant features can be selected, some of which cannot be detected by the radiologist’s eyes.

The overwhelming majority of published radiomics studies focus on oncologic pathology [34]. So far, the application of radiomics in pediatric radiology is rare, generally CT and MRI being the main modalities of examination [34], observing subjects such as cancer [35,36,37], pneumonia analysis [38], or prenatal diagnostics [39]. Up to this point, radiomics has proven its usefulness in a number of studies of the respiratory system, some examples being the ability to distinguish COVID-19 from other non-COVID-19 pneumonias [40,41], attempts to diagnose idiopathic lung fibrosis [42], quantifying the risk for lung cancer [43], or the diagnosis and evaluation of severity and prognosis in COPD patients [44,45].

Tumor genetic and non-genetic heterogeneity arising from variations of vasculature, oxygenation, or metabolism has been previously observed and discussed [46]. The technological advances led to a higher understanding of oncologic pathology and offered the possibility of identifying clinically important phenotypes as targets for new therapeutic interventions [46]. Radiomics could offer opportunities for exploring new possible directions with further potential in clinical practice due to the complex physiopathology of CDH.

The key findings of our study depicted relevant radiomic features that differed between ipsilateral and contralateral lungs. Some of these are shape features that can also be detected by the radiologist. For instance, lung diameter was different (e.g., original_shape_Maximum2DDiameterSlice). These results are in line with other studies that show that the relationship between observed and expected MRI fetal lung volume is a predictor of the need for ECMO therapy [47]. Additional previously published research has also indicated that the diagnosis of thoracic deformities tends to be more prevalent, particularly in cases where extracorporeal membrane oxygenation (ECMO) is required, which further correlates with our results [10]. Other higher-order features, such as original_gldm_LowGrayLevelEmphasis and original_glrlm_LongRunLowGrayLevelEmphasis, show the difference in lung density and aligns with one previous studies which showed a significant reduction in density of the ipsilateral side [24]. These reductions in density observed by means of image voxel relationships should correlate to different degrees of lung hypoplasia and emphysema-like changes and offer potential for future studies with clinical application. However, this requires further research.

For humans, some of these features are hardly comprehensible or visualizable, which underlines the strength of radiomics analysis, as differences in data can be found that would have been missed. The potential of radiomics-assisted feature extraction has been previously discussed and includes applications such as prediction of treatment response and outcomes or tissue identification [48]. Future studies should evaluate whether these features have an impact on clinical courses. This was beyond the scope of this study, as it was meant to be a pilot study.

Previous studies on adults examined with HRCT showed that interobserver variability can vary from fair to moderate or even poor when analyzing lung emphysema [49,50]. When paired with quantitative computed tomography software, the detection and proper grading of emphysema improved by as much as 22% [50]. Previous studies have shown that dose reduction for the examination of children can yield very good results pertaining to the quality of the image [51,52]. However, when compared to HRCT, the quality is undeniably lower, which has the potential for even more interobserver variability. Although not a diagnosis algorithm, radiomics could in such situations be viewed as an additional tool, its purpose being to strengthen the role of the radiologist by guiding clinical judgment or helping to estimate prognoses [30].

In the future, personalized medicine could become a way of adapting treatment strategies to each patient based on their pathology if radiomics proves its potential [48]. Radiologists describe visually the qualitative and quantitative aspects of lung hypoplasia and emphysema-like changes using terms such as “light,” “moderate,” or “advanced,” with the consecutive problem of interobserver variability. A clear threshold would improve the differentiation between borderline cases (such as borderline moderate to advanced), thus offering clear parameters that could in the future be used for further studies and treatment protocols.

A number of severity indicators for CDH in the form of prenatal and postnatal outcomes and severity-predicting factors have been discussed. The most important prenatal factors focus on measurements obtained with ultrasound and MRI [53]. Some of the most important postnatal clinical markers to predict outcome in CDH patients have been gathered by the CDH study group (CDHSG) and are: very low birth weight, absent or low 5-min Apgar score, presence of chromosomal or major cardiac anomaly, and suprasystemic pulmonary hypertension [54]. The Wilford Hall/Santa Rosa Group (WHSR) brought forward the idea that the inability to achieve adequate oxygenation and/or ventilation was associated with a high mortality rate and established a formula based on the difference between the highest Po2 and the highest Pco2 measured in the first 24 h of life, with a result greater than or equal to 0 being a predictor for survival [55]. The increased Pco2 values, which are considered by the CDH EURO Consortium to be a criterion for indicating ECMO therapy, directly relate to the WHSR Formula [6]. Therefore, in our study, we chose status after ECMO therapy as a clinical surrogate for higher disease severity. We found differences in both lungs between ECMO and non-ECMO children with relevant radiomic features that pertain to lung size (original_shape_MinorAxisLength, original_shape_Maximum2DDiameterSlice) and lung density (original_glrlm_LongRunLowGrayLevelEmphasis). This may be a hint that both lungs are affected by severe lung hypoplasia.

Studies, exemplified by the research conducted by Yamoto et al. in 2022, demonstrate that long-term cardio-pulmonary complications not only manifest prominently during early ages but also exhibit the propensity to occur or recur well into adolescence, thereby emphasizing the enduring impact and dynamic nature of these health challenges over an extended developmental timeframe [10]. Consequently, it stands to reason that individuals who have survived congenital diaphragmatic hernia could derive substantial advantages from any advancements in supplementary tools, including radiomics, which could hold the potential of enhancing the ability to stratify the severity of its underlying complex physiopathology or achieve a more precise identification of tissue structures, thereby contributing to more refined and personalized healthcare and treatment for this specific patient population at an early age as well as after follow-ups.

There are several limitations to our study. First, the sample size was small, particularly due to the fact that CDH is a rare disease. However, for a study that we consider explorative, the results look promising. Second, the level of inspiration may play a role in the quality and analysis of features, and since our patients were not sedate, this may introduce a degree of variability. Third, the direct implementation in other centers is limited due to a lack of standardization and limited open-source code and data. Furthermore, when comparing patients that needed ECMO with those that didn’t, one has to consider that the lungs continue to grow and expand in volume from early childhood all the way to adult life, with the majority of our selected patients being of age 1 or higher (with a standard deviation of 51,9 months and a mean deviation of 36.3 months).

## 5. Conclusions

Our study demonstrates the feasibility of radiomic analysis of low-dose chest CT scans after CDH repair and represents morphological differences beyond what is visible to the radiologist. Future studies have to evaluate whether these features can be used as additional prognostic parameters in the follow-up of children after CDH.

Texture analysis using radiomics is still in its early development stage. We consider this to be a pilot study in order to further investigate the relevant medical applications of radiomics-aided feature detection. Radiomics analysis has the potential to become routine in all centers, the purpose being to add quantitative information to the visual assessment made by the radiologist. The extent of this quantitative information could then be further used in elaborating decision trees and protocols to further advance therapy and treatment.

## Figures and Tables

**Table 1:** Selected variables with statistically relevant differences. *n* = number of analyzed lungs.

Compared Variables	Patient Group	Selected Variables	* p * -Value
**Ipsi- vs contralateral**	All (*n* = 144)	original_firstorder_10Percentile	<0.001
		original_gldm_LowGrayLevelEmphasis	<0.001
		original_glrlm_LongRunLowGrayLevelEmphasis	<0.001
		original_shape_Flatness	<0.001
		original_shape_LeastAxisLength	<0.001
		original_shape_Maximum2DDiameterSlice	0.005
**ECMO yes/no**	contralateral-lungs (*n* = 72)	original_shape_MinorAxisLength	0.003
		original_shape_Maximum2DDiameterSlice	0.003
**ECMO yes/no**	ipsilateral-lungs (*n* = 72)	original_glrlm_LongRunLowGrayLevelEmphasis	<0.001

## Data Availability

The data presented in this study are available on request from the corresponding author. The data are not publicly available due to German EHR privacy guidelines.

## Supplementary Materials

The following supporting information can be downloaded at: https://www.mdpi.com/article/10.3390/jcm12247700/s1, S1: Tables of patient characteristics. S2: Pearson correlation coefficient matrix. S3: Plots of Boruta feature selection process for significant differences.
