# Supplementary material for: Radiomics-Assisted Computed Tomography-Based Analysis to Evaluate Lung Morphology Characteristics after Congenital Diaphragmatic Hernia
Source: J Clin Med. 2023 Dec 15;12(24):7700. doi: 10.3390/jcm12247700 (PMC10744187; doi:10.3390/jcm12247700)
Supplement: Supplementary file 1 [file jcm-12-07700-s001.zip › S1. Patient characteristics/tab1_CDH_Ipsi vs contralateral.docx]

| **Comparison of Ipsi- vs contralateral** | **Left lungs** | **Right lungs** | **p** |
| --- | --- | --- | --- |
| n (number of lungs) | 72 | 72 |  |
| Lung Side = right (%) | 50 (69.4) | 22 (30.6) | <0.001 |
| ECMO (mean (SD)) | 0.31 (0.46) | 0.31 (0.46) | 1 |
| CDH (mean (SD)) | 0.00 (0.00) | 1.00 (0.00) | <0.001 |
| original_firstorder_10Percentile (mean (SD)) | -942.42 (48.85) | -976.97 (36.27) | <0.001 |
| original_firstorder_90Percentile (mean (SD)) | -584.44 (98.30) | -646.92 (94.10) | <0.001 |
| original_firstorder_Energy (mean (SD)) | 971829566510.18 (513366176035.17) | 917404537802.71 (493847553741.92) | 0,518 |
| original_firstorder_Entropy (mean (SD)) | 4.33 (0.28) | 4.15 (0.30) | <0.001 |
| original_firstorder_InterquartileRange (mean (SD)) | 186.15 (39.69) | 172.78 (41.51) | 0,05 |
| original_firstorder_Kurtosis (mean (SD)) | 2.84 (0.65) | 3.09 (1.21) | 0,114 |
| original_firstorder_Maximum (mean (SD)) | -408.69 (106.23) | -466.33 (109.09) | 0,002 |
| original_firstorder_Mean (mean (SD)) | -771.04 (71.49) | -822.07 (63.81) | <0.001 |
| original_firstorder_MeanAbsoluteDeviation (mean (SD)) | 108.90 (20.81) | 100.86 (21.14) | 0,023 |
| original_firstorder_Median (mean (SD)) | -781.04 (76.68) | -836.24 (68.75) | <0.001 |
| original_firstorder_Minimum (mean (SD)) | -1024.00 (0.00) | -1023.83 (1.41) | 0,319 |
| original_firstorder_Range (mean (SD)) | 615.31 (106.23) | 557.50 (108.68) | 0,002 |
| original_firstorder_RobustMeanAbsoluteDeviation (mean (SD)) | 77.62 (16.12) | 72.07 (16.86) | 0,045 |
| original_firstorder_RootMeanSquared (mean (SD)) | 783.51 (67.24) | 832.22 (59.48) | <0.001 |
| original_firstorder_Skewness (mean (SD)) | 0.37 (0.39) | 0.59 (0.45) | 0,002 |
| original_firstorder_TotalEnergy (mean (SD)) | 335129647636.37 (354403452053.22) | 322464525705.88 (375390027459.16) | 0,835 |
| original_firstorder_Uniformity (mean (SD)) | 0.06 (0.01) | 0.06 (0.01) | <0.001 |
| original_firstorder_Variance (mean (SD)) | 18804.59 (6706.15) | 16268.08 (6743.54) | 0,025 |
| original_glcm_Autocorrelation (mean (SD)) | 127.42 (66.68) | 86.02 (54.76) | <0.001 |
| original_glcm_ClusterProminence (mean (SD)) | 20452.51 (14381.41) | 15325.19 (13036.90) | 0,027 |
| original_glcm_ClusterShade (mean (SD)) | 148.04 (302.78) | 215.93 (237.78) | 0,137 |
| original_glcm_ClusterTendency (mean (SD)) | 78.52 (30.52) | 66.16 (28.98) | 0,014 |
| original_glcm_Contrast (mean (SD)) | 33.82 (10.52) | 30.22 (11.94) | 0,057 |
| original_glcm_Correlation (mean (SD)) | 0.38 (0.10) | 0.36 (0.10) | 0,218 |
| original_glcm_DifferenceAverage (mean (SD)) | 4.34 (0.73) | 4.04 (0.81) | 0,022 |
| original_glcm_DifferenceEntropy (mean (SD)) | 3.58 (0.24) | 3.50 (0.27) | 0,043 |
| original_glcm_DifferenceVariance (mean (SD)) | 14.02 (4.29) | 12.87 (4.82) | 0,135 |
| original_glcm_Id (mean (SD)) | 0.32 (0.04) | 0.34 (0.05) | 0,008 |
| original_glcm_Idm (mean (SD)) | 0.23 (0.05) | 0.25 (0.05) | 0,008 |
| original_glcm_Idmn (mean (SD)) | 0.95 (0.01) | 0.95 (0.01) | 0,148 |
| original_glcm_Idn (mean (SD)) | 0.86 (0.02) | 0.86 (0.02) | 0,511 |
| original_glcm_Imc1 (mean (SD)) | -0.05 (0.03) | -0.04 (0.03) | 0,764 |
| original_glcm_Imc2 (mean (SD)) | 0.49 (0.11) | 0.47 (0.11) | 0,262 |
| original_glcm_InverseVariance (mean (SD)) | 0.23 (0.03) | 0.24 (0.04) | 0,034 |
| original_glcm_JointAverage (mean (SD)) | 10.42 (2.77) | 8.42 (2.48) | <0.001 |
| original_glcm_JointEnergy (mean (SD)) | 0.00 (0.00) | 0.01 (0.00) | 0,001 |
| original_glcm_JointEntropy (mean (SD)) | 8.39 (0.55) | 8.04 (0.60) | <0.001 |
| original_glcm_MCC (mean (SD)) | 0.43 (0.11) | 0.40 (0.11) | 0,121 |
| original_glcm_MaximumProbability (mean (SD)) | 0.01 (0.01) | 0.03 (0.02) | <0.001 |
| original_glcm_SumAverage (mean (SD)) | 20.85 (5.55) | 16.85 (4.96) | <0.001 |
| original_glcm_SumEntropy (mean (SD)) | 5.07 (0.30) | 4.91 (0.31) | 0,001 |
| original_glcm_SumSquares (mean (SD)) | 28.09 (9.93) | 24.09 (10.00) | 0,018 |
| original_gldm_DependenceEntropy (mean (SD)) | 6.91 (0.24) | 6.84 (0.23) | 0,064 |
| original_gldm_DependenceNonUniformity (mean (SD)) | 262341.83 (102592.76) | 202472.80 (91330.34) | <0.001 |
| original_gldm_DependenceNonUniformityNormalized (mean (SD)) | 0.18 (0.03) | 0.16 (0.03) | 0,001 |
| original_gldm_DependenceVariance (mean (SD)) | 5.51 (3.62) | 7.94 (4.73) | 0,001 |
| original_gldm_GrayLevelNonUniformity (mean (SD)) | 88743.44 (49325.33) | 87765.48 (54818.79) | 0,911 |
| original_gldm_GrayLevelVariance (mean (SD)) | 30.04 (10.72) | 25.91 (10.76) | 0,023 |
| original_gldm_HighGrayLevelEmphasis (mean (SD)) | 152.28 (73.69) | 107.16 (62.42) | <0.001 |
| original_gldm_LargeDependenceEmphasis (mean (SD)) | 17.04 (11.31) | 22.47 (14.36) | 0,013 |
| original_gldm_LargeDependenceHighGrayLevelEmphasis (mean (SD)) | 1517.97 (1930.08) | 1030.27 (1089.06) | 0,064 |
| original_gldm_LargeDependenceLowGrayLevelEmphasis (mean (SD)) | 4.99 (3.79) | 9.56 (6.61) | <0.001 |
| original_gldm_LowGrayLevelEmphasis (mean (SD)) | 0.07 (0.03) | 0.11 (0.05) | <0.001 |
| original_gldm_SmallDependenceEmphasis (mean (SD)) | 0.29 (0.05) | 0.26 (0.06) | 0,005 |
| original_gldm_SmallDependenceHighGrayLevelEmphasis (mean (SD)) | 59.34 (30.21) | 43.40 (28.73) | 0,001 |
| original_gldm_SmallDependenceLowGrayLevelEmphasis (mean (SD)) | 0.01 (0.00) | 0.01 (0.00) | 0,008 |
| original_glrlm_GrayLevelNonUniformity (mean (SD)) | 78919.79 (42248.35) | 75489.62 (45881.49) | 0,641 |
| original_glrlm_GrayLevelNonUniformityNormalized (mean (SD)) | 0.06 (0.01) | 0.06 (0.01) | 0,001 |
| original_glrlm_GrayLevelVariance (mean (SD)) | 30.02 (10.55) | 25.94 (10.57) | 0,022 |
| original_glrlm_HighGrayLevelRunEmphasis (mean (SD)) | 156.06 (74.02) | 111.35 (62.54) | <0.001 |
| original_glrlm_LongRunEmphasis (mean (SD)) | 1.36 (0.26) | 1.44 (0.34) | 0,076 |
| original_glrlm_LongRunHighGrayLevelEmphasis (mean (SD)) | 192.39 (102.22) | 136.32 (76.75) | <0.001 |
| original_glrlm_LongRunLowGrayLevelEmphasis (mean (SD)) | 0.13 (0.08) | 0.24 (0.13) | <0.001 |
| original_glrlm_LowGrayLevelRunEmphasis (mean (SD)) | 0.06 (0.03) | 0.09 (0.04) | <0.001 |
| original_glrlm_RunEntropy (mean (SD)) | 4.79 (0.23) | 4.67 (0.23) | 0,003 |
| original_glrlm_RunLengthNonUniformity (mean (SD)) | 1146639.91 (462056.18) | 938715.86 (424278.03) | 0,006 |
| original_glrlm_RunLengthNonUniformityNormalized (mean (SD)) | 0.85 (0.04) | 0.83 (0.05) | 0,004 |
| original_glrlm_RunPercentage (mean (SD)) | 0.91 (0.03) | 0.90 (0.03) | 0,006 |
| original_glrlm_RunVariance (mean (SD)) | 0.14 (0.13) | 0.18 (0.17) | 0,091 |
| original_glrlm_ShortRunEmphasis (mean (SD)) | 0.94 (0.02) | 0.93 (0.02) | 0,008 |
| original_glrlm_ShortRunHighGrayLevelEmphasis (mean (SD)) | 148.83 (70.71) | 106.35 (60.38) | <0.001 |
| original_glrlm_ShortRunLowGrayLevelEmphasis (mean (SD)) | 0.05 (0.03) | 0.08 (0.03) | <0.001 |
| original_glszm_GrayLevelNonUniformity (mean (SD)) | 21958.53 (9210.95) | 18049.42 (8309.78) | 0,008 |
| original_glszm_GrayLevelNonUniformityNormalized (mean (SD)) | 0.05 (0.01) | 0.05 (0.01) | 0,004 |
| original_glszm_GrayLevelVariance (mean (SD)) | 36.33 (11.26) | 31.27 (11.20) | 0,008 |
| original_glszm_HighGrayLevelZoneEmphasis (mean (SD)) | 201.20 (75.44) | 160.17 (61.62) | <0.001 |
| original_glszm_LargeAreaEmphasis (mean (SD)) | 41203.92 (147693.28) | 81226.37 (232815.41) | 0,22 |
| original_glszm_LargeAreaHighGrayLevelEmphasis (mean (SD)) | 3659650.44 (25239795.92) | 3043344.12 (18932454.94) | 0,869 |
| original_glszm_LargeAreaLowGrayLevelEmphasis (mean (SD)) | 5273.03 (11728.57) | 20361.22 (42846.31) | 0,005 |
| original_glszm_LowGrayLevelZoneEmphasis (mean (SD)) | 0.04 (0.02) | 0.05 (0.02) | 0,006 |
| original_glszm_SizeZoneNonUniformity (mean (SD)) | 176533.38 (79583.71) | 135155.88 (70741.91) | 0,001 |
| original_glszm_SizeZoneNonUniformityNormalized (mean (SD)) | 0.38 (0.02) | 0.38 (0.03) | 0,317 |
| original_glszm_SmallAreaEmphasis (mean (SD)) | 0.64 (0.02) | 0.64 (0.03) | 0,323 |
| original_glszm_SmallAreaHighGrayLevelEmphasis (mean (SD)) | 139.99 (51.53) | 113.70 (44.24) | 0,001 |
| original_glszm_SmallAreaLowGrayLevelEmphasis (mean (SD)) | 0.02 (0.01) | 0.02 (0.01) | 0,049 |
| original_glszm_ZoneEntropy (mean (SD)) | 6.67 (0.21) | 6.54 (0.22) | <0.001 |
| original_glszm_ZonePercentage (mean (SD)) | 0.32 (0.07) | 0.29 (0.07) | 0,004 |
| original_glszm_ZoneVariance (mean (SD)) | 41191.61 (147679.41) | 81209.11 (232787.96) | 0,22 |
| original_ngtdm_Busyness (mean (SD)) | 1558.18 (1404.64) | 2124.48 (1776.24) | 0,036 |
| original_ngtdm_Coarseness (mean (SD)) | 0.00 (0.00) | 0.00 (0.00) | 0,215 |
| original_ngtdm_Complexity (mean (SD)) | 781.55 (334.67) | 655.80 (380.68) | 0,037 |
| original_ngtdm_Contrast (mean (SD)) | 0.31 (0.09) | 0.30 (0.10) | 0,794 |
| original_ngtdm_Strength (mean (SD)) | 0.00 (0.00) | 0.00 (0.00) | 0,6 |
| original_shape_Compactness1 (mean (SD)) | 0.01 (0.00) | 0.01 (0.00) | 0,006 |
| original_shape_Compactness2 (mean (SD)) | 0.01 (0.01) | 0.02 (0.01) | 0,008 |
| original_shape_Elongation (mean (SD)) | 0.80 (0.10) | 0.73 (0.12) | 0,001 |
| original_shape_Flatness (mean (SD)) | 0.53 (0.08) | 0.45 (0.07) | <0.001 |
| original_shape_LeastAxisLength (mean (SD)) | 67.07 (15.25) | 57.89 (14.54) | <0.001 |
| original_shape_MajorAxisLength (mean (SD)) | 128.78 (35.84) | 130.54 (36.85) | 0,771 |
| original_shape_Maximum2DDiameterColumn (mean (SD)) | 156.41 (41.71) | 153.13 (43.87) | 0,646 |
| original_shape_Maximum2DDiameterRow (mean (SD)) | 152.51 (39.90) | 151.53 (39.63) | 0,882 |
| original_shape_Maximum2DDiameterSlice (mean (SD)) | 118.72 (26.09) | 106.51 (25.44) | 0,005 |
| original_shape_Maximum3DDiameter (mean (SD)) | 170.60 (43.73) | 164.04 (44.45) | 0,373 |
| original_shape_MeshVolume (mean (SD)) | 500804.42 (462525.48) | 439816.87 (469694.48) | 0,434 |
| original_shape_MinorAxisLength (mean (SD)) | 101.17 (23.41) | 94.12 (24.21) | 0,078 |
| original_shape_SphericalDisproportion (mean (SD)) | 5.21 (1.63) | 4.54 (1.35) | 0,008 |
| original_shape_Sphericity (mean (SD)) | 0.21 (0.06) | 0.24 (0.06) | 0,006 |
| original_shape_SurfaceArea (mean (SD)) | 140333.30 (70877.89) | 109517.46 (59287.69) | 0,005 |
| original_shape_SurfaceVolumeRatio (mean (SD)) | 0.37 (0.16) | 0.34 (0.16) | 0,351 |
| original_shape_VoxelVolume (mean (SD)) | 495928.07 (458208.61) | 435367.43 (465427.80) | 0,433 |
