# Supplementary material for: Radiomics-Assisted Computed Tomography-Based Analysis to Evaluate Lung Morphology Characteristics after Congenital Diaphragmatic Hernia
Source: J Clin Med. 2023 Dec 15;12(24):7700. doi: 10.3390/jcm12247700 (PMC10744187; doi:10.3390/jcm12247700)
Supplement: Supplementary file 1 [file jcm-12-07700-s001.zip › S1. Patient characteristics/tab1_Comparison between left and right.docx]

| **Comparison between left and right** | **Left lungs** | **Right lungs** | **p** |
| --- | --- | --- | --- |
| n (number of lungs) | 72 | 72 |  |
| Lung Side = right (%) | 0 (0.0) | 72 (100.0) | <0.001 |
| ECMO (mean (SD)) | 0.31 (0.46) | 0.31 (0.46) | 1 |
| CDH (mean (SD)) | 0.69 (0.46) | 0.31 (0.46) | <0.001 |
| original_firstorder_10Percentile (mean (SD)) | -965.05 (42.94) | -954.33 (49.04) | 0,165 |
| original_firstorder_90Percentile (mean (SD)) | -621.18 (99.29) | -610.18 (102.84) | 0,515 |
| original_firstorder_Energy (mean (SD)) | 881240130804.38 (457597604680.56) | 1007993973508.51 (539805666791.05) | 0,131 |
| original_firstorder_Entropy (mean (SD)) | 4.23 (0.30) | 4.25 (0.31) | 0,752 |
| original_firstorder_InterquartileRange (mean (SD)) | 179.64 (41.01) | 179.29 (41.31) | 0,959 |
| original_firstorder_Kurtosis (mean (SD)) | 2.98 (0.96) | 2.95 (1.00) | 0,89 |
| original_firstorder_Maximum (mean (SD)) | -443.26 (109.56) | -431.76 (113.13) | 0,537 |
| original_firstorder_Mean (mean (SD)) | -802.76 (69.47) | -790.35 (74.82) | 0,304 |
| original_firstorder_MeanAbsoluteDeviation (mean (SD)) | 104.90 (21.17) | 104.86 (21.55) | 0,992 |
| original_firstorder_Median (mean (SD)) | -815.96 (74.20) | -801.32 (80.84) | 0,26 |
| original_firstorder_Minimum (mean (SD)) | -1024.00 (0.00) | -1023.83 (1.41) | 0,319 |
| original_firstorder_Range (mean (SD)) | 580.74 (109.56) | 592.07 (112.79) | 0,542 |
| original_firstorder_RobustMeanAbsoluteDeviation (mean (SD)) | 74.92 (16.67) | 74.77 (16.79) | 0,959 |
| original_firstorder_RootMeanSquared (mean (SD)) | 813.95 (65.14) | 801.78 (70.32) | 0,283 |
| original_firstorder_Skewness (mean (SD)) | 0.52 (0.41) | 0.44 (0.45) | 0,242 |
| original_firstorder_TotalEnergy (mean (SD)) | 305619498629.33 (333131130096.53) | 351974674712.93 (393109308449.31) | 0,447 |
| original_firstorder_Uniformity (mean (SD)) | 0.06 (0.01) | 0.06 (0.01) | 0,734 |
| original_firstorder_Variance (mean (SD)) | 17529.62 (6868.16) | 17543.05 (6821.90) | 0,991 |
| original_glcm_Autocorrelation (mean (SD)) | 101.39 (60.82) | 112.05 (67.51) | 0,321 |
| original_glcm_ClusterProminence (mean (SD)) | 17642.47 (14085.87) | 18135.23 (13841.27) | 0,833 |
| original_glcm_ClusterShade (mean (SD)) | 210.68 (265.38) | 153.29 (280.10) | 0,209 |
| original_glcm_ClusterTendency (mean (SD)) | 71.89 (30.01) | 72.79 (30.79) | 0,861 |
| original_glcm_Contrast (mean (SD)) | 32.19 (11.60) | 31.85 (11.19) | 0,859 |
| original_glcm_Correlation (mean (SD)) | 0.37 (0.10) | 0.38 (0.10) | 0,737 |
| original_glcm_DifferenceAverage (mean (SD)) | 4.19 (0.79) | 4.18 (0.78) | 0,931 |
| original_glcm_DifferenceEntropy (mean (SD)) | 3.54 (0.26) | 3.54 (0.25) | 0,881 |
| original_glcm_DifferenceVariance (mean (SD)) | 13.56 (4.72) | 13.33 (4.48) | 0,76 |
| original_glcm_Id (mean (SD)) | 0.33 (0.05) | 0.33 (0.04) | 0,875 |
| original_glcm_Idm (mean (SD)) | 0.24 (0.05) | 0.24 (0.05) | 0,854 |
| original_glcm_Idmn (mean (SD)) | 0.95 (0.01) | 0.95 (0.01) | 0,531 |
| original_glcm_Idn (mean (SD)) | 0.86 (0.02) | 0.86 (0.02) | 0,648 |
| original_glcm_Imc1 (mean (SD)) | -0.04 (0.03) | -0.04 (0.03) | 0,846 |
| original_glcm_Imc2 (mean (SD)) | 0.48 (0.11) | 0.49 (0.12) | 0,676 |
| original_glcm_InverseVariance (mean (SD)) | 0.23 (0.04) | 0.23 (0.04) | 0,94 |
| original_glcm_JointAverage (mean (SD)) | 9.18 (2.71) | 9.67 (2.90) | 0,296 |
| original_glcm_JointEnergy (mean (SD)) | 0.01 (0.00) | 0.01 (0.00) | 0,658 |
| original_glcm_JointEntropy (mean (SD)) | 8.20 (0.60) | 8.23 (0.60) | 0,763 |
| original_glcm_MCC (mean (SD)) | 0.41 (0.10) | 0.43 (0.12) | 0,356 |
| original_glcm_MaximumProbability (mean (SD)) | 0.02 (0.02) | 0.02 (0.01) | 0,347 |
| original_glcm_SumAverage (mean (SD)) | 18.36 (5.41) | 19.34 (5.81) | 0,296 |
| original_glcm_SumEntropy (mean (SD)) | 4.99 (0.31) | 5.00 (0.32) | 0,829 |
| original_glcm_SumSquares (mean (SD)) | 26.02 (10.14) | 26.16 (10.19) | 0,935 |
| original_gldm_DependenceEntropy (mean (SD)) | 6.87 (0.23) | 6.88 (0.25) | 0,859 |
| original_gldm_DependenceNonUniformity (mean (SD)) | 212762.40 (90250.25) | 252052.24 (108425.42) | 0,019 |
| original_gldm_DependenceNonUniformityNormalized (mean (SD)) | 0.17 (0.03) | 0.17 (0.03) | 0,613 |
| original_gldm_DependenceVariance (mean (SD)) | 6.83 (4.61) | 6.62 (4.15) | 0,776 |
| original_gldm_GrayLevelNonUniformity (mean (SD)) | 82049.72 (47867.98) | 94459.21 (55399.89) | 0,153 |
| original_gldm_GrayLevelVariance (mean (SD)) | 27.95 (10.97) | 27.99 (10.91) | 0,982 |
| original_gldm_HighGrayLevelEmphasis (mean (SD)) | 124.31 (68.05) | 135.13 (75.29) | 0,367 |
| original_gldm_LargeDependenceEmphasis (mean (SD)) | 20.13 (14.34) | 19.37 (11.96) | 0,733 |
| original_gldm_LargeDependenceHighGrayLevelEmphasis (mean (SD)) | 1166.43 (1112.68) | 1381.82 (1941.73) | 0,415 |
| original_gldm_LargeDependenceLowGrayLevelEmphasis (mean (SD)) | 7.58 (6.17) | 6.97 (5.50) | 0,537 |
| original_gldm_LowGrayLevelEmphasis (mean (SD)) | 0.10 (0.05) | 0.08 (0.05) | 0,195 |
| original_gldm_SmallDependenceEmphasis (mean (SD)) | 0.28 (0.05) | 0.28 (0.06) | 0,86 |
| original_gldm_SmallDependenceHighGrayLevelEmphasis (mean (SD)) | 50.08 (29.22) | 52.66 (31.78) | 0,613 |
| original_gldm_SmallDependenceLowGrayLevelEmphasis (mean (SD)) | 0.01 (0.00) | 0.01 (0.00) | 0,355 |
| original_glrlm_GrayLevelNonUniformity (mean (SD)) | 71372.69 (39868.84) | 83036.72 (47302.15) | 0,112 |
| original_glrlm_GrayLevelNonUniformityNormalized (mean (SD)) | 0.06 (0.01) | 0.06 (0.01) | 0,815 |
| original_glrlm_GrayLevelVariance (mean (SD)) | 28.00 (10.82) | 27.96 (10.70) | 0,982 |
| original_glrlm_HighGrayLevelRunEmphasis (mean (SD)) | 128.32 (68.06) | 139.09 (75.58) | 0,371 |
| original_glrlm_LongRunEmphasis (mean (SD)) | 1.41 (0.34) | 1.39 (0.26) | 0,763 |
| original_glrlm_LongRunHighGrayLevelEmphasis (mean (SD)) | 156.51 (83.35) | 172.19 (104.22) | 0,321 |
| original_glrlm_LongRunLowGrayLevelEmphasis (mean (SD)) | 0.19 (0.12) | 0.18 (0.11) | 0,37 |
| original_glrlm_LowGrayLevelRunEmphasis (mean (SD)) | 0.08 (0.04) | 0.07 (0.04) | 0,178 |
| original_glrlm_RunEntropy (mean (SD)) | 4.73 (0.23) | 4.73 (0.25) | 0,891 |
| original_glrlm_RunLengthNonUniformity (mean (SD)) | 958049.63 (406728.36) | 1127306.14 (485257.49) | 0,025 |
| original_glrlm_RunLengthNonUniformityNormalized (mean (SD)) | 0.83 (0.05) | 0.84 (0.04) | 0,665 |
| original_glrlm_RunPercentage (mean (SD)) | 0.90 (0.04) | 0.90 (0.03) | 0,71 |
| original_glrlm_RunVariance (mean (SD)) | 0.17 (0.17) | 0.16 (0.13) | 0,812 |
| original_glrlm_ShortRunEmphasis (mean (SD)) | 0.93 (0.02) | 0.93 (0.02) | 0,672 |
| original_glrlm_ShortRunHighGrayLevelEmphasis (mean (SD)) | 122.60 (65.50) | 132.58 (72.25) | 0,387 |
| original_glrlm_ShortRunLowGrayLevelEmphasis (mean (SD)) | 0.07 (0.03) | 0.06 (0.03) | 0,185 |
| original_glszm_GrayLevelNonUniformity (mean (SD)) | 18346.78 (7977.98) | 21661.18 (9613.75) | 0,026 |
| original_glszm_GrayLevelNonUniformityNormalized (mean (SD)) | 0.05 (0.01) | 0.05 (0.01) | 0,938 |
| original_glszm_GrayLevelVariance (mean (SD)) | 33.76 (11.66) | 33.85 (11.36) | 0,962 |
| original_glszm_HighGrayLevelZoneEmphasis (mean (SD)) | 176.48 (67.72) | 184.89 (75.63) | 0,483 |
| original_glszm_LargeAreaEmphasis (mean (SD)) | 62431.24 (222230.26) | 59999.05 (165647.85) | 0,941 |
| original_glszm_LargeAreaHighGrayLevelEmphasis (mean (SD)) | 2826990.93 (18935142.37) | 3876003.62 (25230540.22) | 0,778 |
| original_glszm_LargeAreaLowGrayLevelEmphasis (mean (SD)) | 14192.90 (39002.01) | 11441.35 (23744.47) | 0,61 |
| original_glszm_LowGrayLevelZoneEmphasis (mean (SD)) | 0.04 (0.02) | 0.04 (0.02) | 0,478 |
| original_glszm_SizeZoneNonUniformity (mean (SD)) | 144166.00 (71589.60) | 167523.26 (82488.77) | 0,072 |
| original_glszm_SizeZoneNonUniformityNormalized (mean (SD)) | 0.38 (0.03) | 0.38 (0.03) | 0,53 |
| original_glszm_SmallAreaEmphasis (mean (SD)) | 0.64 (0.02) | 0.64 (0.02) | 0,51 |
| original_glszm_SmallAreaHighGrayLevelEmphasis (mean (SD)) | 124.63 (47.48) | 129.06 (51.95) | 0,594 |
| original_glszm_SmallAreaLowGrayLevelEmphasis (mean (SD)) | 0.02 (0.01) | 0.02 (0.01) | 0,588 |
| original_glszm_ZoneEntropy (mean (SD)) | 6.60 (0.23) | 6.61 (0.22) | 0,736 |
| original_glszm_ZonePercentage (mean (SD)) | 0.30 (0.07) | 0.31 (0.07) | 0,836 |
| original_glszm_ZoneVariance (mean (SD)) | 62415.50 (222201.59) | 59985.22 (165634.78) | 0,941 |
| original_ngtdm_Busyness (mean (SD)) | 1774.17 (1434.03) | 1908.49 (1795.84) | 0,621 |
| original_ngtdm_Coarseness (mean (SD)) | 0.00 (0.00) | 0.00 (0.00) | 0,271 |
| original_ngtdm_Complexity (mean (SD)) | 710.56 (368.29) | 726.79 (359.40) | 0,789 |
| original_ngtdm_Contrast (mean (SD)) | 0.31 (0.10) | 0.30 (0.10) | 0,589 |
| original_ngtdm_Strength (mean (SD)) | 0.00 (0.00) | 0.00 (0.00) | 0,788 |
| original_shape_Compactness1 (mean (SD)) | 0.01 (0.00) | 0.01 (0.00) | 0,046 |
| original_shape_Compactness2 (mean (SD)) | 0.02 (0.01) | 0.01 (0.01) | 0,098 |
| original_shape_Elongation (mean (SD)) | 0.74 (0.12) | 0.79 (0.11) | 0,007 |
| original_shape_Flatness (mean (SD)) | 0.45 (0.07) | 0.53 (0.08) | <0.001 |
| original_shape_LeastAxisLength (mean (SD)) | 58.61 (15.69) | 66.36 (14.49) | 0,002 |
| original_shape_MajorAxisLength (mean (SD)) | 130.78 (34.24) | 128.54 (38.33) | 0,711 |
| original_shape_Maximum2DDiameterColumn (mean (SD)) | 152.53 (43.39) | 157.01 (42.15) | 0,531 |
| original_shape_Maximum2DDiameterRow (mean (SD)) | 150.88 (38.05) | 153.17 (41.39) | 0,73 |
| original_shape_Maximum2DDiameterSlice (mean (SD)) | 108.62 (26.09) | 116.61 (26.27) | 0,069 |
| original_shape_Maximum3DDiameter (mean (SD)) | 162.32 (43.88) | 172.32 (43.97) | 0,174 |
| original_shape_MeshVolume (mean (SD)) | 433437.74 (429531.65) | 507183.55 (499173.01) | 0,344 |
| original_shape_MinorAxisLength (mean (SD)) | 95.21 (22.47) | 100.08 (25.34) | 0,225 |
| original_shape_SphericalDisproportion (mean (SD)) | 4.61 (1.41) | 5.13 (1.61) | 0,042 |
| original_shape_Sphericity (mean (SD)) | 0.23 (0.06) | 0.21 (0.06) | 0,04 |
| original_shape_SurfaceArea (mean (SD)) | 111277.57 (57757.07) | 138573.18 (72846.07) | 0,014 |
| original_shape_SurfaceVolumeRatio (mean (SD)) | 0.35 (0.16) | 0.36 (0.16) | 0,506 |
| original_shape_VoxelVolume (mean (SD)) | 429376.53 (426103.57) | 501918.97 (494171.30) | 0,347 |
