# Supplementary material for: Radiomics-Assisted Computed Tomography-Based Analysis to Evaluate Lung Morphology Characteristics after Congenital Diaphragmatic Hernia
Source: J Clin Med. 2023 Dec 15;12(24):7700. doi: 10.3390/jcm12247700 (PMC10744187; doi:10.3390/jcm12247700)
Supplement: Supplementary file 1 [file jcm-12-07700-s001.zip › S1. Patient characteristics/tab1_ECMO.docx]

| **Comparison of ECMO/non-ECMO Patients** | **Non-ECMO** | **ECMO** | **p** |
| --- | --- | --- | --- |
| n (number of lungs) | 100 | 44 |  |
| Lung side = right (%) | 50 (50.0) | 22 (50.0) | 1 |
| ECMO (mean (SD)) | 0.00 (0.00) | 1.00 (0.00) | <0.001 |
| CDH (mean (SD)) | 0.50 (0.50) | 0.50 (0.51) | 1 |
| original_firstorder_10Percentile (mean (SD)) | -955.43 (46.28) | -969.39 (45.17) | 0,095 |
| original_firstorder_90Percentile (mean (SD)) | -603.11 (100.85) | -644.25 (96.03) | 0,024 |
| original_firstorder_Energy (mean (SD)) | 877145308228.93 (444747920664.96) | 1097961924718.98 (591844241048.47) | 0,015 |
| original_firstorder_Entropy (mean (SD)) | 4.28 (0.29) | 4.14 (0.32) | 0,014 |
| original_firstorder_InterquartileRange (mean (SD)) | 184.45 (39.54) | 168.14 (42.49) | 0,027 |
| original_firstorder_Kurtosis (mean (SD)) | 2.81 (0.66) | 3.32 (1.41) | 0,003 |
| original_firstorder_Maximum (mean (SD)) | -434.84 (110.82) | -443.59 (112.85) | 0,665 |
| original_firstorder_Mean (mean (SD)) | -787.29 (72.97) | -817.60 (66.57) | 0,02 |
| original_firstorder_MeanAbsoluteDeviation (mean (SD)) | 107.21 (20.73) | 99.58 (21.83) | 0,047 |
| original_firstorder_Median (mean (SD)) | -798.13 (78.38) | -832.52 (71.25) | 0,014 |
| original_firstorder_Minimum (mean (SD)) | -1023.88 (1.20) | -1024.00 (0.00) | 0,509 |
| original_firstorder_Range (mean (SD)) | 589.04 (110.56) | 580.41 (112.85) | 0,669 |
| original_firstorder_RobustMeanAbsoluteDeviation (mean (SD)) | 76.85 (16.10) | 70.30 (17.22) | 0,029 |
| original_firstorder_RootMeanSquared (mean (SD)) | 799.13 (68.48) | 827.72 (62.58) | 0,019 |
| original_firstorder_Skewness (mean (SD)) | 0.41 (0.39) | 0.64 (0.48) | 0,003 |
| original_firstorder_TotalEnergy (mean (SD)) | 262060145907.53 (262973999379.22) | 480471952042.93 (497076732447.00) | 0,001 |
| original_firstorder_Uniformity (mean (SD)) | 0.06 (0.01) | 0.07 (0.02) | 0,005 |
| original_firstorder_Variance (mean (SD)) | 18148.18 (6820.80) | 16145.77 (6690.99) | 0,105 |
| original_glcm_Autocorrelation (mean (SD)) | 114.38 (66.20) | 89.31 (56.48) | 0,031 |
| original_glcm_ClusterProminence (mean (SD)) | 18591.89 (13722.71) | 16291.04 (14381.41) | 0,363 |
| original_glcm_ClusterShade (mean (SD)) | 158.54 (270.83) | 235.26 (274.84) | 0,121 |
| original_glcm_ClusterTendency (mean (SD)) | 75.07 (29.67) | 66.15 (31.13) | 0,104 |
| original_glcm_Contrast (mean (SD)) | 33.42 (11.83) | 28.85 (9.57) | 0,026 |
| original_glcm_Correlation (mean (SD)) | 0.37 (0.08) | 0.37 (0.13) | 0,946 |
| original_glcm_DifferenceAverage (mean (SD)) | 4.30 (0.76) | 3.92 (0.77) | 0,007 |
| original_glcm_DifferenceEntropy (mean (SD)) | 3.58 (0.22) | 3.45 (0.31) | 0,008 |
| original_glcm_DifferenceVariance (mean (SD)) | 13.87 (4.75) | 12.48 (4.07) | 0,094 |
| original_glcm_Id (mean (SD)) | 0.32 (0.03) | 0.35 (0.06) | 0,003 |
| original_glcm_Idm (mean (SD)) | 0.23 (0.04) | 0.26 (0.07) | 0,003 |
| original_glcm_Idmn (mean (SD)) | 0.95 (0.01) | 0.96 (0.01) | 0,021 |
| original_glcm_Idn (mean (SD)) | 0.86 (0.01) | 0.87 (0.02) | 0,007 |
| original_glcm_Imc1 (mean (SD)) | -0.04 (0.01) | -0.05 (0.05) | 0,144 |
| original_glcm_Imc2 (mean (SD)) | 0.48 (0.09) | 0.48 (0.15) | 0,822 |
| original_glcm_InverseVariance (mean (SD)) | 0.23 (0.03) | 0.25 (0.04) | 0,002 |
| original_glcm_JointAverage (mean (SD)) | 9.80 (2.84) | 8.58 (2.56) | 0,016 |
| original_glcm_JointEnergy (mean (SD)) | 0.00 (0.00) | 0.01 (0.00) | 0,002 |
| original_glcm_JointEntropy (mean (SD)) | 8.31 (0.55) | 8.00 (0.65) | 0,004 |
| original_glcm_MCC (mean (SD)) | 0.42 (0.09) | 0.41 (0.15) | 0,651 |
| original_glcm_MaximumProbability (mean (SD)) | 0.02 (0.01) | 0.02 (0.02) | 0,083 |
| original_glcm_SumAverage (mean (SD)) | 19.59 (5.69) | 17.16 (5.12) | 0,016 |
| original_glcm_SumEntropy (mean (SD)) | 5.03 (0.30) | 4.90 (0.33) | 0,018 |
| original_glcm_SumSquares (mean (SD)) | 27.12 (10.13) | 23.75 (9.84) | 0,065 |
| original_gldm_DependenceEntropy (mean (SD)) | 6.88 (0.19) | 6.87 (0.31) | 0,959 |
| original_gldm_DependenceNonUniformity (mean (SD)) | 227550.93 (93329.40) | 243444.57 (117978.19) | 0,388 |
| original_gldm_DependenceNonUniformityNormalized (mean (SD)) | 0.18 (0.03) | 0.16 (0.03) | 0,001 |
| original_gldm_DependenceVariance (mean (SD)) | 6.24 (3.45) | 7.82 (5.86) | 0,046 |
| original_gldm_GrayLevelNonUniformity (mean (SD)) | 80091.57 (43592.11) | 106806.48 (64013.92) | 0,004 |
| original_gldm_GrayLevelVariance (mean (SD)) | 28.95 (10.89) | 25.75 (10.71) | 0,104 |
| original_gldm_HighGrayLevelEmphasis (mean (SD)) | 138.14 (73.83) | 110.59 (63.35) | 0,033 |
| original_gldm_LargeDependenceEmphasis (mean (SD)) | 17.95 (7.42) | 23.85 (20.61) | 0,013 |
| original_gldm_LargeDependenceHighGrayLevelEmphasis (mean (SD)) | 1173.62 (553.16) | 1502.54 (2743.55) | 0,251 |
| original_gldm_LargeDependenceLowGrayLevelEmphasis (mean (SD)) | 7.03 (5.95) | 7.82 (5.59) | 0,459 |
| original_gldm_LowGrayLevelEmphasis (mean (SD)) | 0.08 (0.05) | 0.10 (0.05) | 0,037 |
| original_gldm_SmallDependenceEmphasis (mean (SD)) | 0.28 (0.05) | 0.26 (0.05) | 0,004 |
| original_gldm_SmallDependenceHighGrayLevelEmphasis (mean (SD)) | 55.14 (31.99) | 42.79 (24.87) | 0,024 |
| original_gldm_SmallDependenceLowGrayLevelEmphasis (mean (SD)) | 0.01 (0.00) | 0.01 (0.01) | 0,024 |
| original_glrlm_GrayLevelNonUniformity (mean (SD)) | 70593.63 (36582.55) | 92229.87 (54946.51) | 0,006 |
| original_glrlm_GrayLevelNonUniformityNormalized (mean (SD)) | 0.06 (0.01) | 0.06 (0.01) | 0,005 |
| original_glrlm_GrayLevelVariance (mean (SD)) | 28.90 (10.75) | 25.86 (10.47) | 0,117 |
| original_glrlm_HighGrayLevelRunEmphasis (mean (SD)) | 141.92 (73.74) | 115.04 (64.39) | 0,038 |
| original_glrlm_LongRunEmphasis (mean (SD)) | 1.36 (0.12) | 1.50 (0.50) | 0,011 |
| original_glrlm_LongRunHighGrayLevelEmphasis (mean (SD)) | 170.03 (86.11) | 151.46 (110.88) | 0,278 |
| original_glrlm_LongRunLowGrayLevelEmphasis (mean (SD)) | 0.18 (0.12) | 0.21 (0.11) | 0,124 |
| original_glrlm_LowGrayLevelRunEmphasis (mean (SD)) | 0.07 (0.04) | 0.09 (0.04) | 0,017 |
| original_glrlm_RunEntropy (mean (SD)) | 4.75 (0.23) | 4.68 (0.26) | 0,126 |
| original_glrlm_RunLengthNonUniformity (mean (SD)) | 1001836.14 (408874.98) | 1135500.04 (536977.21) | 0,104 |
| original_glrlm_RunLengthNonUniformityNormalized (mean (SD)) | 0.84 (0.03) | 0.82 (0.06) | 0,003 |
| original_glrlm_RunPercentage (mean (SD)) | 0.91 (0.02) | 0.89 (0.05) | 0,005 |
| original_glrlm_RunVariance (mean (SD)) | 0.14 (0.06) | 0.21 (0.26) | 0,015 |
| original_glrlm_ShortRunEmphasis (mean (SD)) | 0.93 (0.01) | 0.92 (0.03) | 0,004 |
| original_glrlm_ShortRunHighGrayLevelEmphasis (mean (SD)) | 135.75 (70.98) | 109.02 (60.64) | 0,031 |
| original_glrlm_ShortRunLowGrayLevelEmphasis (mean (SD)) | 0.06 (0.03) | 0.07 (0.04) | 0,01 |
| original_glszm_GrayLevelNonUniformity (mean (SD)) | 19477.01 (8256.14) | 21201.64 (10385.03) | 0,289 |
| original_glszm_GrayLevelNonUniformityNormalized (mean (SD)) | 0.05 (0.01) | 0.05 (0.01) | 0,028 |
| original_glszm_GrayLevelVariance (mean (SD)) | 34.60 (11.40) | 31.99 (11.56) | 0,211 |
| original_glszm_HighGrayLevelZoneEmphasis (mean (SD)) | 186.02 (72.33) | 168.57 (69.39) | 0,179 |
| original_glszm_LargeAreaEmphasis (mean (SD)) | 30124.46 (72772.51) | 131875.79 (327576.86) | 0,004 |
| original_glszm_LargeAreaHighGrayLevelEmphasis (mean (SD)) | 561373.65 (1430864.30) | 9692687.34 (39749997.87) | 0,023 |
| original_glszm_LargeAreaLowGrayLevelEmphasis (mean (SD)) | 11655.93 (33868.94) | 15456.20 (28243.05) | 0,516 |
| original_glszm_LowGrayLevelZoneEmphasis (mean (SD)) | 0.04 (0.02) | 0.05 (0.02) | 0,013 |
| original_glszm_SizeZoneNonUniformity (mean (SD)) | 153805.23 (79716.29) | 160479.62 (74108.23) | 0,637 |
| original_glszm_SizeZoneNonUniformityNormalized (mean (SD)) | 0.38 (0.03) | 0.39 (0.03) | 0,044 |
| original_glszm_SmallAreaEmphasis (mean (SD)) | 0.64 (0.02) | 0.65 (0.02) | 0,038 |
| original_glszm_SmallAreaHighGrayLevelEmphasis (mean (SD)) | 130.01 (50.81) | 119.65 (46.62) | 0,25 |
| original_glszm_SmallAreaLowGrayLevelEmphasis (mean (SD)) | 0.02 (0.01) | 0.03 (0.01) | 0,017 |
| original_glszm_ZoneEntropy (mean (SD)) | 6.64 (0.20) | 6.53 (0.27) | 0,007 |
| original_glszm_ZonePercentage (mean (SD)) | 0.32 (0.07) | 0.28 (0.07) | 0,002 |
| original_glszm_ZoneVariance (mean (SD)) | 30112.76 (72767.93) | 131854.00 (327538.38) | 0,004 |
| original_ngtdm_Busyness (mean (SD)) | 1673.61 (1393.88) | 2222.51 (2012.07) | 0,061 |
| original_ngtdm_Coarseness (mean (SD)) | 0.00 (0.00) | 0.00 (0.00) | 0,286 |
| original_ngtdm_Complexity (mean (SD)) | 725.73 (365.87) | 702.64 (359.00) | 0,726 |
| original_ngtdm_Contrast (mean (SD)) | 0.32 (0.09) | 0.27 (0.09) | 0,013 |
| original_ngtdm_Strength (mean (SD)) | 0.00 (0.00) | 0.00 (0.00) | 0,676 |
| original_shape_Compactness1 (mean (SD)) | 0.01 (0.00) | 0.01 (0.00) | 0,168 |
| original_shape_Compactness2 (mean (SD)) | 0.01 (0.01) | 0.02 (0.02) | 0,119 |
| original_shape_Elongation (mean (SD)) | 0.77 (0.12) | 0.77 (0.11) | 0,976 |
| original_shape_Flatness (mean (SD)) | 0.50 (0.09) | 0.47 (0.08) | 0,098 |
| original_shape_LeastAxisLength (mean (SD)) | 60.54 (12.84) | 66.91 (19.88) | 0,023 |
| original_shape_MajorAxisLength (mean (SD)) | 123.47 (29.74) | 143.72 (45.15) | 0,002 |
| original_shape_Maximum2DDiameterColumn (mean (SD)) | 148.14 (35.50) | 169.84 (53.14) | 0,005 |
| original_shape_Maximum2DDiameterRow (mean (SD)) | 144.91 (31.48) | 168.18 (50.57) | 0,001 |
| original_shape_Maximum2DDiameterSlice (mean (SD)) | 107.75 (19.51) | 123.67 (35.50) | 0,001 |
| original_shape_Maximum3DDiameter (mean (SD)) | 159.92 (36.18) | 184.14 (54.99) | 0,002 |
| original_shape_MeshVolume (mean (SD)) | 388187.47 (345285.17) | 656954.24 (628328.78) | 0,001 |
| original_shape_MinorAxisLength (mean (SD)) | 93.12 (19.01) | 107.92 (30.45) | 0,001 |
| original_shape_SphericalDisproportion (mean (SD)) | 4.96 (1.55) | 4.67 (1.47) | 0,287 |
| original_shape_Sphericity (mean (SD)) | 0.22 (0.06) | 0.23 (0.07) | 0,19 |
| original_shape_SurfaceArea (mean (SD)) | 116843.06 (57967.52) | 143294.27 (81610.60) | 0,028 |
| original_shape_SurfaceVolumeRatio (mean (SD)) | 0.37 (0.14) | 0.33 (0.19) | 0,186 |
| original_shape_VoxelVolume (mean (SD)) | 383774.42 (341151.56) | 651723.49 (623255.53) | 0,001 |
