# Supplementary figures and images for: Radiomics-Assisted Computed Tomography-Based Analysis to Evaluate Lung Morphology Characteristics after Congenital Diaphragmatic Hernia
Source: J Clin Med. 2023 Dec 15;12(24):7700. doi: 10.3390/jcm12247700 (PMC10744187; doi:10.3390/jcm12247700)

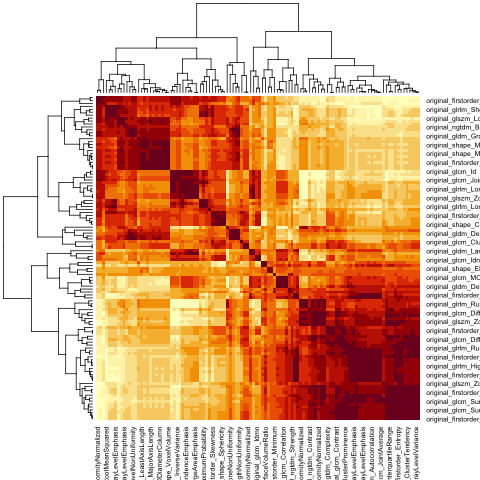

Supplement: Supplementary file 1 [file jcm-12-07700-s001.zip › S2. Pearson correlation coefficient matrix/data_virlan_small_features.cor.png]

Variable Importance

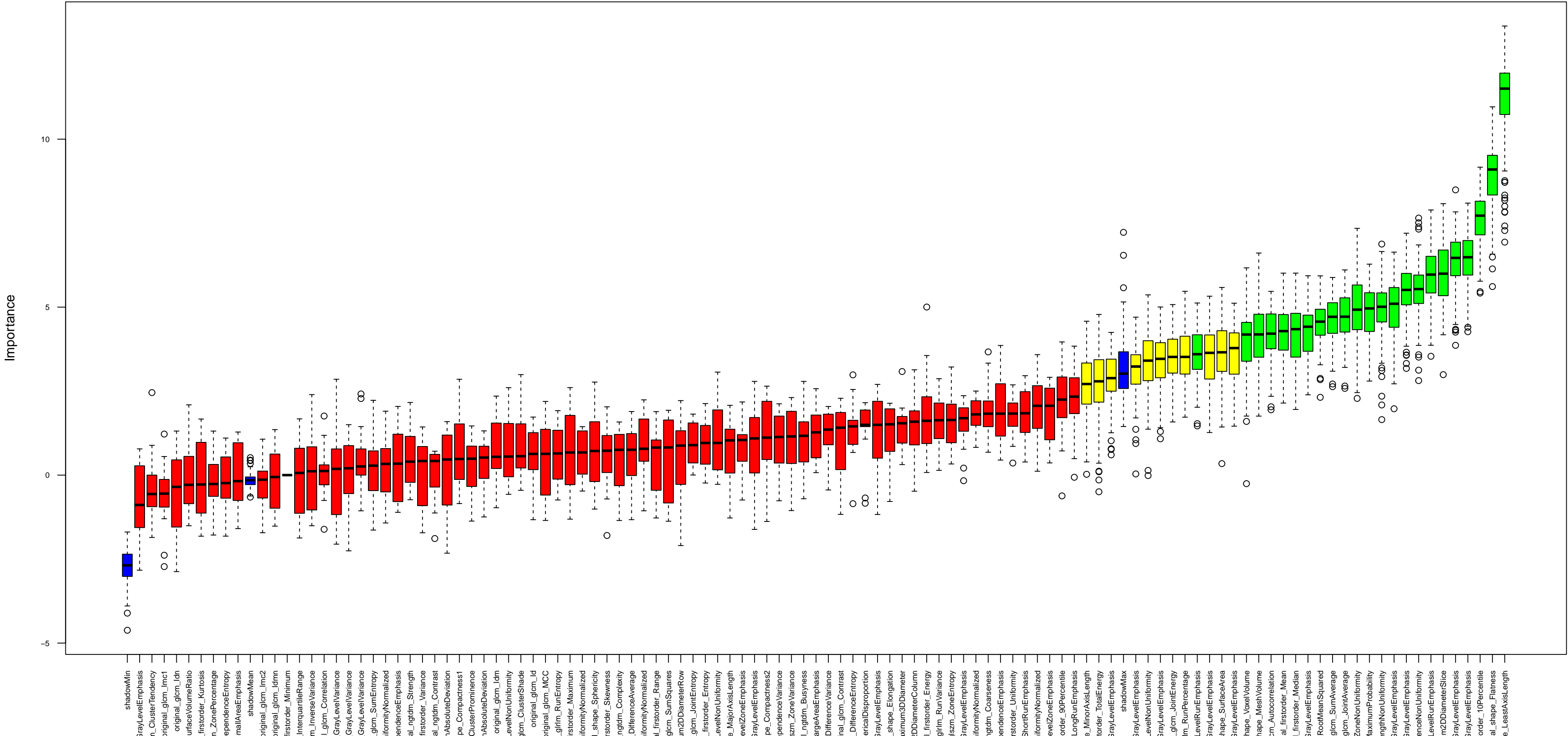

Supplement: Supplementary file 1 [file jcm-12-07700-s001.zip › S3. Plots of Boruta feature selection process for significant differences/CDH_importance.pdf]

Variable Importance

Importance

-5 0 5 10

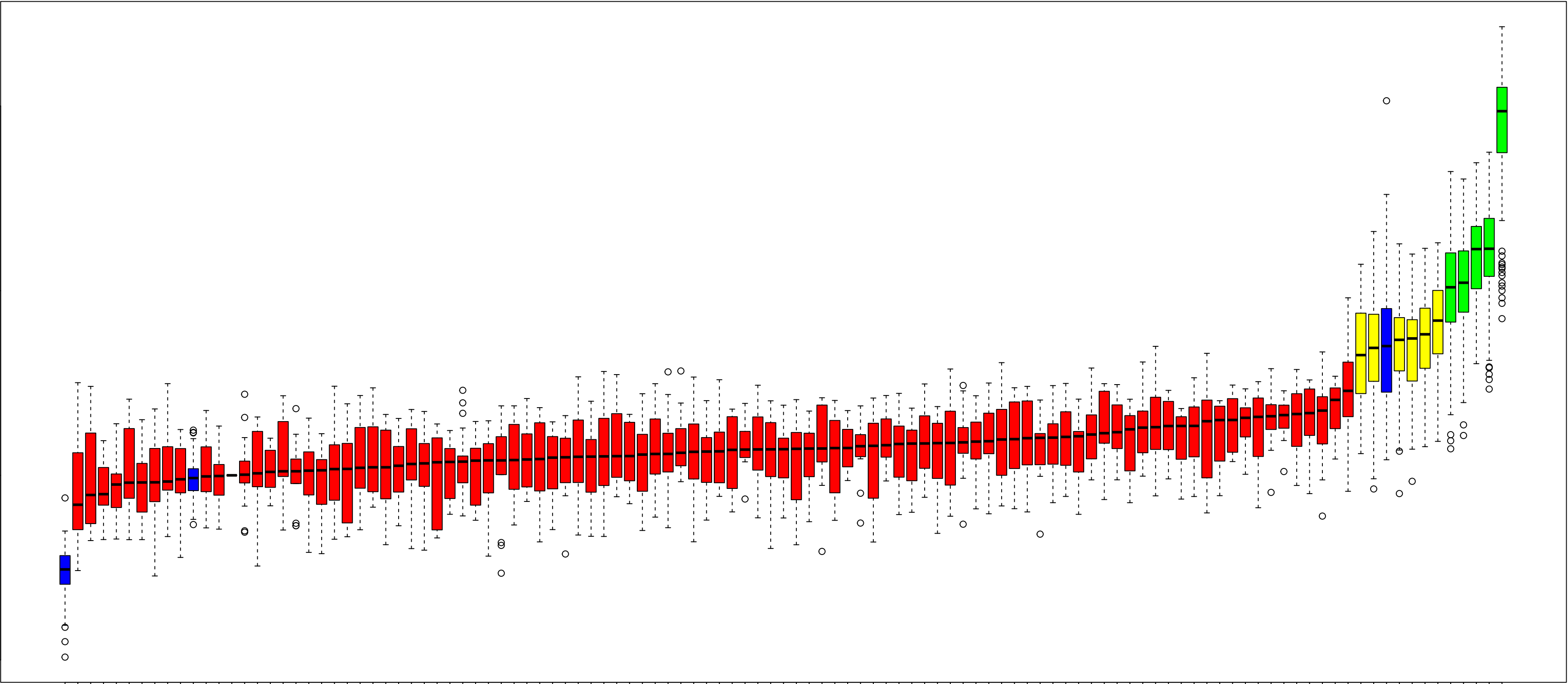

Supplement: Supplementary file 1 [file jcm-12-07700-s001.zip › S3. Plots of Boruta feature selection process for significant differences/ECMO_Importance.pdf]
